# Octascope: A Lightweight Pre-Trained Model for Optical Coherence Tomography

**DOI:** 10.1109/access.2025.3595838

**Published:** 2025-08-05

**Authors:** HAOYANG CUI, CHEN WANG, PAUL CALLE, YUNLONG LIU, QINGHAO ZHANG, SINARO LY, JUSTIN REYNOLDS, FENG YAN, KE ZHANG, RONGHAO LIU, JUNYUAN LIU, KAR-MING FUNG, ZHONGXIN YU, AJAY JAIN, QINGGONG TANG, CHONGLE PAN

**Affiliations:** 1 School of Computer Science, Gallogly College of Engineering, The University of Oklahoma, Norman, OK 73019, USA; 2 Stephenson School of Biomedical Engineering, The University of Oklahoma, Norman, OK 73019, USA; 3 Department of Pathology, University of Oklahoma Health Sciences Center, Oklahoma City, OK 73104, USA; 4 Stephenson Cancer Center, University of Oklahoma Health Sciences Center, Oklahoma City, OK 73104, USA; 5 Department of Urology, University of Oklahoma Health Sciences Center, Oklahoma City, OK 73104, USA; 6 Department of Surgery, University of Oklahoma Health Sciences Center, Oklahoma City, OK 73104, USA

**Keywords:** Deep learning, domain-specific, foundation model, lightweight, Octascope, OCT medical imaging, transfer learning

## Abstract

Optical coherence tomography (OCT) imaging enables high resolution visualization of sub-surface tissue microstructures. However, OCT image analysis using deep learning is hampered by limited diverse training data to meet performance requirements and high inference latency for real-time applications. To address these challenges, we developed Octascope, a lightweight domain-specific convolutional neural network (CNN) - based model designed for OCT image analysis. Octascope was pre-trained using a curriculum learning approach, which involves sequential training, first on natural images (ImageNet), then on OCT images from retinal, abdominal, and renal tissues, to progressively acquire transferable knowledge. This multi-domain pre-training enables Octascope to generalize across varied tissue types. In two downstream tasks, Octascope demonstrated notable improvements in predictive accuracy compared to alternative approaches. In the epidural tissue detection task, our method surpassed single-task learning with fine-tuning by 9.13% and OCT-specific transfer learning by 5.95% in accuracy. Octascope outperformed VGG16 and ResNet50 by 5.36% and 6.66% in a retinal diagnosis task, respectively. In comparison to a Transformer-based OCT foundation model - RETFound, Octascope delivered 2 to 4.4 times faster inference speed with slightly better predictive accuracies in both downstream tasks. Octascope represented a significant advancement for OCT image analysis by providing an effective balance between computational efficiency and diagnostic accuracy for real-time clinical applications.

## I. INTRODUCTION

Machine learning models can acquire knowledge to perform a task from three sources: the training data for the task at hand, the human expert insights imparted via induction biases and regularizations, and knowledge gained through transfer learning from other related tasks. Both the training data and human expertise are often scarce and costly to acquire for many applications. Consequently, transfer learning has emerged in recent years as an important strategy for enhancing the performance of machine learning models [28], [29], [30]. The performance gain and generalization capability obtained from transfer learning are scalable with the number of related tasks and the amount of training data used for the pre-training. A model obtained through extensive pre-training on a wide variety of related tasks within an application domain can be referred to as a foundation model for that domain [37], [38]. Foundation models typically have a large number of parameters to fully assimilate the knowledge obtained in the extensive pre-training. Generalizable knowledge allows foundation models to excel in many downstream tasks in their domain without using extensive task-specific training data.

The application domains for foundation models should be carefully defined to include tasks that allow positive knowledge transfer among them and exclude tasks susceptible to negative transfer. Foundation models have been developed for many general-purpose application domains, including the Generative Pre-trained Transformer (GPT) for text generation [1], Segment Anything Model (SAM) for image segmentation [2], and GattacaNet for pan-disease predictive genomics [3]. In the medical imaging field, foundation models have been developed for MRI [4], X-ray [5], and ultrasound [6]. By developing a foundation model for each medical imaging modality, an adequate performance can be obtained for new diagnosis by fine-tuning the corresponding foundation model using a small set of training data for a downstream task.

While the development of large-scale foundation models has gained substantial attention, there is also a growing trend toward building lightweight models optimized for real-world clinical use. These models aim to strike a balance between diagnostic performance and computational efficiency, making them more accessible for deployment in constrained environments. For example, recent frameworks like VFMGL [40] and LlaVA-Rad [41] demonstrate that it is possible to transfer knowledge from large models to smaller, task-specific networks without compromising accuracy. These efforts highlight an emerging direction that prioritizes practicality and accessibility.

Optical Coherence Tomography (OCT) is a non-invasive imaging technique that is analogous to ultrasound imaging, but measures backscattered light from optical scattering media instead of ultrasound. OCT provides micro-meter-resolution, cross-sectional images from within biological tissues. It has been widely used to image the retina for the diagnosis and monitoring of many ocular conditions in ophthalmology. RETFound [34] is a foundation model for OCT and color fundus photography (CFP) images of retina trained from ImageNet-1k first then on the Moorfields diabetic image dataset (MEH-MIDAS) and other public datasets (Kaggle EyePACs [25] and Kermany [15]). MEH-MIDAS included the complete ocular imaging records of 37401 patients with diabetes who were at the Moorfields Eye Hospital. RETFound achieved an Area Under Receiver Operating Characteristic curve (AUROC) of 0.799 for predicting the development of neovascular (‘wet’) age-related macular degeneration (AMD) in a patient’s contralateral (‘fellow’) eye using OCT images. This was statistically significantly higher (P < 0.001, two-sided t-test) than the AUROC of 0.783 by SSL-Retinal (Self-supervised Learning), which is a model trained from scratch using only retinal OCT images with self-supervised learning. The pre-training of RETFound with related retinal datasets not only increased the predictive performance but also reduced the amount of labeled data needed for the downstream tasks. A RETFound fine-tuned model using only 10% of the labeled training data achieved a higher accuracy in predicting heart failure and myocardial infarction than other models, including SL-ImageNet, SSL-ImageNet and SSL-Retinal, which were trained using all the labeled training data. OCTCube [39] is a recently proposed domain-specific 3D foundation model that extensively leverages the volumetric structure of OCT data to improve diagnostic generalization. It is pre-trained on 26,605 OCT volumes (over 1.6 million 2D slices) using 3D masked autoencoders and optimized with FlashAttention to mitigate GPU memory limitations. OCTCube has demonstrated superior performance over 2D models in predicting retinal and systemic diseases across datasets, devices, and modalities, including through a novel contrastive OCT-infrared (IR) alignment framework. While foundation models such as RETFound and OCTCube have shown promise in OCT-based diagnosis, they remain largely specialized for retinal data and entail high computational costs which limit their practical use in time-sensitive or non-retinal clinical settings.

In recent years, OCT has been extended to imaging many non-retinal tissues, not only for disease diagnosis but also for imaging-guided procedures. For example, OCT has been used to image cancer of human mucosa [7] and to image the kidneys to assess their viability for transplantations [8]. In our previous work, by integrating a forward-viewing OCT probe within an endoscope, we have demonstrated real-time surgical guidance in epidural anesthesia [9], percutaneous nephrostomy [10], and renal carcinoma biopsy [11]. Thus far, the OCT imaging data from these applications have been processed using traditional imaging analysis methods [12] or single-task deep learning models [13]. The existing OCT foundation models, including RETFound and SSL-Retinal, were not pre-trained using any non-retina OCT data.

To address these gaps, we built Octascope, a lightweight convolution neural network (CNN)-based model designed to support OCT image analysis across multiple tissue types. Our hypothesis is that pre-training on OCT images from varied tissues can enhance generalization and transferability, even without large-scale model capacity. Octascope is trained using a curriculum learning strategy, where the model is first exposed to natural images (ImageNet), followed by progressive domain adaptation to retinal, renal [11], and abdominal [14] OCT datasets with 449,300 2D images. Regarding Octascope, we use the term ‘foundation model’ in a domain-specific context: a pre-trained model tailored for OCT that supports generalization across tissues and tasks. We refer to it as a domain-specific foundation model within the OCT modality.

The main objectives of this study are to design and develop a lightweight, pre-trained model tailored for OCT imaging that can generalize effectively across multiple tissue types and clinical tasks. We aim to improve prediction accuracy in scenarios with limited labeled data by leveraging curriculum learning: sequentially transferring knowledge from large-scale natural image datasets to domain-specific OCT datasets. In addition, we seek to benchmark the performance and computational efficiency of our proposed model, Octascope, against RETFound, a state-of-the-art transformer-based foundation model.

The key contributions of this work include the introduction of Octascope, a CNN trained on a diverse multi-tissue OCT dataset using a curriculum learning framework. We demonstrate its effectiveness on two downstream tasks: epidural tissue classification and retinal disease diagnosis where it outperformed several baseline models while achieving significantly faster inference. Furthermore, we provide an in-depth evaluation of various transfer learning strategies, including fine-tuning, feature extraction, and progressive freezing. To enhance interpretability, we also present Grad-CAM visualizations that offer insights into the model’s decision-making across different tissue domains.

## II. DATASETS

### A. PRE-TRAINING DATA FOR THE DEVELOPMENT OF OCTASCOPE

Three pre-training datasets were used to build Octascope. The first dataset, Retinal OCT, comprises 109,309 OCT images categorized into four classes: choroidal neovascularization (CNV), diabetic macular edema (DME), drusen, and normal [15]. This dataset underwent a rigorous tiered grading system, in which multiple layers of trained graders with increasing expertise verified and corrected the image labels. The second dataset, Veress Needle, contains images of five tissue types (subcutaneous fat, intestine, muscle, abdominal space, and skin) collected from eight pig specimens. Each tissue type included 1,000 2D-OCT cross-sections, resulting in a total of 40,000 images [14]. The third dataset, Human Kidney [11], consisted of samples from five human subjects with malignant renal carcinoma, along with normal renal tissues, including the cortex, medulla, calyx, fat, and pelvis [11]. This dataset contained 10,000 images per tissue type per subject. The 2D-OCT cross sectional images from Veress Needle and Human Kidney were derived from their corresponding original 3D OCT volumes.

### B. DOWNSTREAM TASKS FOR FINE-TUNING AND EVALUATION OF OCTASCOPE

Two downstream tasks, namely, the epidural tissue detection task and the OCTDL [16] retinal diagnosis task, were employed to benchmark the predictive performance and computational efficiency of the domain-specific foundation models. The epidural tissue detection task involved identification of the spinal tissues from forward-view endoscopic OCT images to guide the placement of Tuohy needle in the epidural space of the spine for anesthesia [9]. The epidural tissue detection dataset contained four types of spinal tissue (fat, ligament, flavum, and spinal cord) from eight pig subjects, with 1,000 images per tissue type per subject, except for the seventh subject which had 700 spinal cord images. The OCTDL retinal diagnosis task aimed to diagnose eight retinal conditions using the OCTDL, which comprised 2064 labeled B-scan OCT images of retina with the following conditions: Age-related Macular Degeneration (AMD), diabetic macular edema (DME), vitreomacular interface disease (VID), epiretinal membrane (ERM), normal cases (Normal), retinal artery occlusion (RAO), and retinal vein occlusion (RVO). The number of scans for each condition were: 1,231, 147, 155, 332, 22, 101, and 76. There was no overlap between the DME and normal images in the OCTDL retinal diagnosis dataset and those in the Retinal OCT dataset used for pre-training. The fat tissues in both the pre-training and epidural detection datasets were treated as separate categories for classification due to the distinct characteristics of the fat tissues presented in different organs.

## III. METHODS

### A. PRE-TRAINING DATASET

Our models were developed using two convolutional neural network (CNN) architectures, InceptionV3 [18] and ResNet50 [19], under the TensorFlow framework [27]. We constructed two lightweight foundation models, OCT Transfer Learning (OCT-TL), and Octascope, and a non-foundation model, ImageNet-TL. The CNN models for OCT-TL were initialized with random weights. The CNN models for Octascope and ImageNet-TL imported the pre-trained ImageNet [31] weights from Keras [32]. A ‘GlobalAveragPooling2D’ layer was attached to the last layer of both InceptionV3 and ResNet50.

Retinal OCT, Veress Needle, and Human Kidney datasets were merged into a single unified pre-training dataset with 15 distinct classes. The Veress Needle dataset had eight subjects; each fold corresponded to one subject. The Human Kidney and Retinal OCT datasets were also split into eight folds. This pre-training dataset was used to train the CNN models for both OCT-TL and Octascope. ImageNet-TL was not pre-trained on any OCT data. All the training and evaluations were conducted using NVIDIA GeForce RTX 4090 graphics cards on an Ubuntu Linux workstation.

### B. NESTED CROSS-VALIDATION (NCV) FOR OCTASCOPE DEVELOPMENT AND DOWNSTREAM TASKS

We utilized nested cross-validation (NCV) [17], [46] for training and evaluating both the domain-specific foundation models and the epidural tissue detection. NCV consists of three nested loops arranged from outermost to innermost: the cross-testing (CT) loop, Hyperparameter Optimization (HPO) loop, and cross-validation (CV) loop, as shown in Algorithm. Based on empirical experience, we defined 36 hyperparameter configurations with learning rates ranging from 0.01 to 0.0001, epochs from 20 to 50, and batch sizes from 16 to 64. The process began by splitting the data into k folds. In the CT loop, the fold *i* was reserved as the test set. Each predefined hyperparameter configuration was evaluated within the HPO loop. In each configuration, the CV loop designated one of the folds as the validation set, whereas the remaining k−2 folds formed the training set. After computing validation performance for all CV iterations, the average performance for the specified hyperparameter configuration was calculated. Once all configurations were evaluated in the HPO loop, the configuration the highest average validation performance was selected. Using this optimal configuration, a model was then trained on k−1 folds and evaluated on the held-out test fold; this process was repeated for each test set. Finally, we calculated the accuracy and standard errors of the test performance across all folds, providing an estimation of the model’s performance when encountering new unseen subjects. In Octascope and OCT-TL development, after the completion of NCV, we trained the models again with all folds of training data using the hyperparameter configurations that produced the highest accuracy in the CV loop for Octascope and OCT-TL.

### C. ADAPTATION TO DOWNSTREAM TASKS

Two transfer learning methods were evaluated for adapting ImageNet-TL, OCT-TL, and Octascope to the downstream tasks: feature extraction and fine-tuning. The feature extraction method preserved the learned representations by freezing all convolutional layers in the CNN architecture and disabling their trainability. This forced the model to operate in the inference mode for these layers, effectively using them as fixed feature extractors. Only the final classification layer (Soft-Max layer) remained trainable, allowing the model to adapt its decision boundary to the new task using pre-learned features. The fine-tuning method retrained the entire model on the downstream tasks. To mitigate catastrophic forgetting and preserve the beneficial features learned during pre-training, we implemented a fine-tuning process with a significantly reduced learning rate (0.0001 or 0.00001), depending on the specific training and validation accuracy. Stochastic Gradient Descent (SGD) was used as the optimizer for transfer learning [24]. RETFound was adapted to the two downstream tasks by fine-tuning its published weights exclusively for the OCT data.

The epidural tissue detection task contained eight subjects in eight data folds, and the test performance of all adapted pre-trained models was evaluated for each subject using the NCV procedure. The test performance of RETFound was also benchmarked using the same NCV procedure. In the OCTDL retinal diagnosis task, we adopted the data partitioning protocol from the original study, which divided images at the patient level into training, validation, and test sets. Strict patient-level separation was maintained across all sets to ensure that all images from a single patient, regardless of the retinal condition, were assigned to only one set. This rigorous separation prevented any potential data leakage that could artificially inflate the predictive accuracy of the model. The image size for both downstream tasks under Octascope was 216 × 330 pixels. In experiments with RETFound, we reshaped the size to 224 × 224 for its structural requirements.

### D. EVALUATION METRICS

We report the per-class precision, recall, and $F_{1}$ score in the retinal diagnosis task to evaluate class-wise performance to address the imbalanced nature of OCTDL. These are defined below:

(1) $$Precision=\frac{TP}{TP+FP}$$

(2) $$Recall=\frac{TP}{TP+FN}$$

(3) $$F_{1}score=2\cdot \frac{Precision\cdot Recall}{Precision+Recall}$$

where TP, FP, and FN denote true positives, false positives, and false negatives, respectively. We also report macroaveraged F1-score, computed as the mean of $F_{1}$ score across all classes.

**Table:** 

Algorithm 1 Nested Cross-Validation
$$\begin{array}{cc} \; & \;k:\text{number of folds}\\ \; & \;\text{Divide the data into k folds}\\ \; & \;∕\mathrm{Cross}\text{-}\mathrm{testing}\;\mathrm{loop}∕\\ \; & \;\mathrm{for}\;\text{subject}\;i\;\text{in the}\;k\;\text{subjects do}\\ \; & \;\;\;\text{Hold out subject}\;i\;\text{in the test set}\\ \; & \;\;\;∕\mathrm{HPO}\;\mathrm{loop}∕\\ \; & \;\;\;\mathrm{for}\;\text{each hyperparameter configuration do}\\ \; & \;\;\;\;\;∕\mathrm{Corss}\text{-}\mathrm{validation}\;\mathrm{loop}∕\\ \; & \;\;\;\;\;\mathrm{for}\;\text{subject}\;j\;\text{in the remaining}\;k-1\;\text{subjects do}\\ \; & \;\;\;\;\;\;\;\text{Use subject}\;j\;\text{as the validations set}\\ \; & \;\;\;\;\;\;\;\text{Train the DNN model using the remaining}\;k-2\;\text{sub-}\\ \; & \;\text{jects as training set}\\ \; & \;\;\;\;\;\;\;\text{Benchmark the validation performance using subject j}\\ \; & \;\;\;\;\;\mathrm{end}\;\mathrm{for}\\ \; & \;\;\;\;\;\text{Estimate the mean validation performance}\\ \; & \;\;\;\mathrm{end}\;\mathrm{for}\\ \; & \;\;\;\text{Select the best hyperparameter configuration for each test}\\ \; & \;\text{subject based on the mean validation performance}\\ \; & \;\;\;\text{Train the model with the selected hyperparameter config-}\\ \; & \;\text{uration using all the remaining}\;k-1\;\text{subjects}\\ \; & \;\;\;\text{Benchmark test performance using subject}\;i\\ \; & \;\mathrm{end}\;\mathrm{for}\\ \; & \;\text{Calculate the accuracy and standard error for the test} \end{array}$$

## IV. RESULTS

### A. COMPARISON OF MODELS FOR THE EPIDURAL TISSUE DETECTION TASK

**FIGURE 3.**
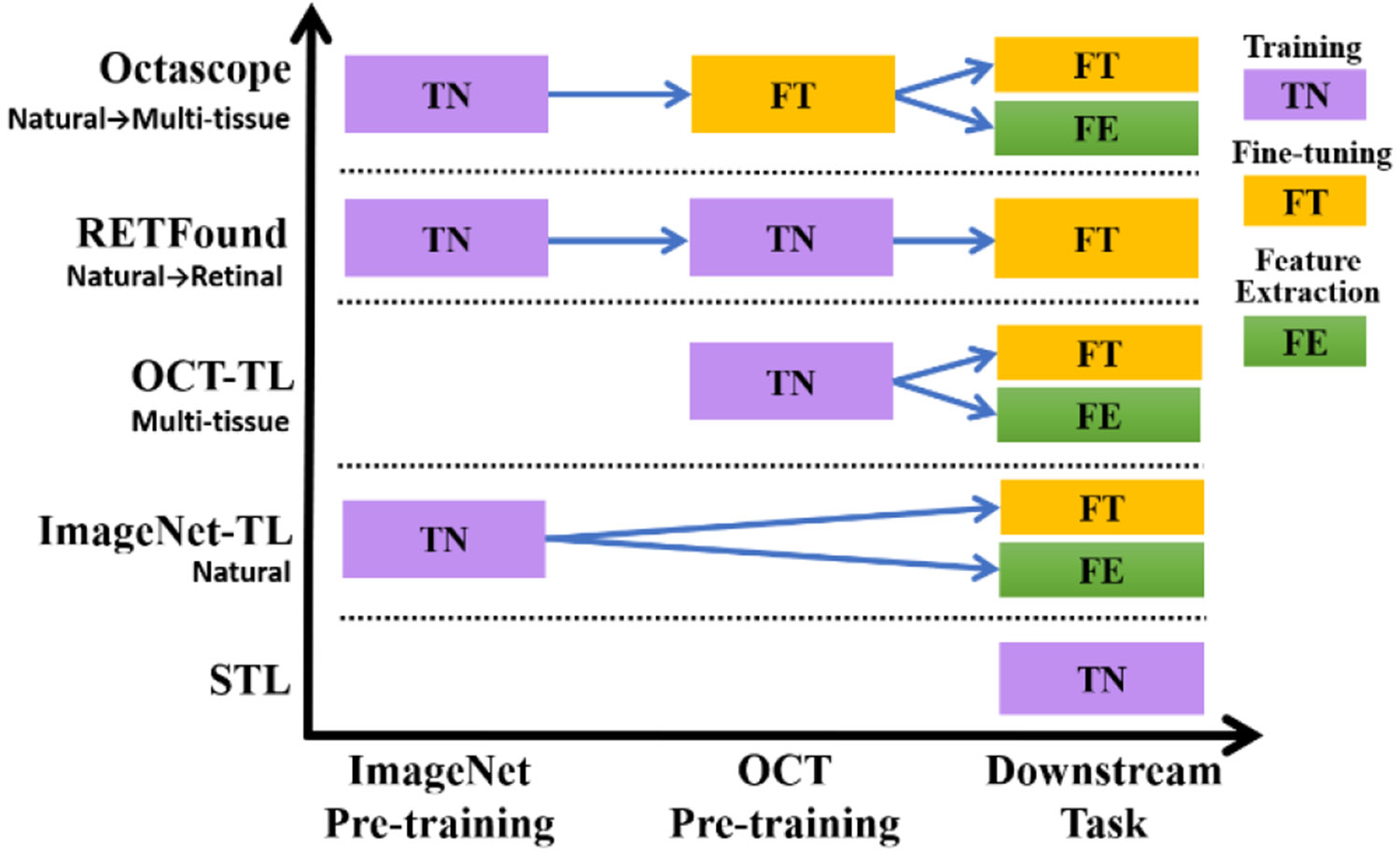
Overview of training strategies across different base models and training stages. Base models (listed vertically) showed different training procedures through three sequential stages (horizontally): ImageNet pre-training, OCT pre-training, and adaptation to the downstream task. Purple blocks (TN) indicate training from scratch, yellow blocks (FT) represent fine-tuning, and green blocks (FE) indicate feature extraction. It demonstrates how sequential pre-training stages can be strategically designed to bridge the gap between natural image and medical imaging.

We compared five approaches for the epidural tissue detection task: STL (single-task learning), ImageNet-TL (ImageNet transfer learning), OCT-TL (OCT transfer learning), Octascope, and RETFound (Figure 3). These approaches had different processes for pre-training and different strategies for downstream adaptation. We chose InceptionV3 as the CNN architecture for Octascope because it outperformed ResNet50 in all approaches. For example, it delivered an average accuracy improvement of 10.02% under both ImageNet-TL and OCT-TL with fine-tuning. In addition, it demonstrated approximately 4.5 times faster inference speed compared with ResNet50.

Table 1 presented the predictive accuracy of the different training strategies for the epidural tissue detection task. Of the two downstream adaptation methods, fine-tuning consistently outperformed feature extraction regardless of whether the models were initialized with random or pre-trained weights. The extent of performance improvement ranged from a 1.39% increase with ImageNet-TL, to a more substantial 4.96% improvement with OCT-TL (P=0.0068, paired t-test), and finally, the largest enhancement of 5.38% with Octascope (P=0.0494). These increasing amounts of performance gain suggested that the benefits of fine-tuning become more pronounced with more extensive pre-training.

The STL established a baseline performance level, achieving an average accuracy of 59.50 ± 1.67% across eight subjects. The ImageNet-TL model demonstrated improvements over STL in both feature extraction (60.87 ± 2.63%) and fine-tuning (62.26 ± 3.49%). This enhancement suggested that, despite the substantial differences between natural and medical images, the knowledge learned from ImageNet’s diverse natural dataset contributed positively to this tissue classification task.

When applying fine-tuning for downstream adaptation, Octascope achieved an accuracy and standard error (SE) of 68.63 ± 2.93% compared to OCT-TL’s 62.68 ± 2.54% (P=0.0032). The improvement in the test performance of Octascope was obtained consistently across all eight subjects, with particularly substantial gains observed in S1 (71.22% vs 66.28%) and S4 (73.68% vs 60.30%). While OCT-TL used only OCT images for pre-training, Octascope first learned from the diverse natural images in ImageNet before its pre-training using OCT images. This highlighted the advantages of curriculum learning [35], which enabled a model to first acquire general knowledge before developing specialized expertise. While OCT-TL used only OCT images for pre-training, Octascope first learned from the diverse natural images in ImageNet before its subsequent pre-training using OCT images. This sequential learning strategy allowed Octascope to benefit from the rich visual diversity and broad contextual features, which are often lacking in domain-specific medical datasets. Such a learning paradigm enhances model generalization and robustness, as evidenced by Octascope’s superior performance in downstream tasks compared with models pre-trained solely on OCT data.

**FIGURE 4.**
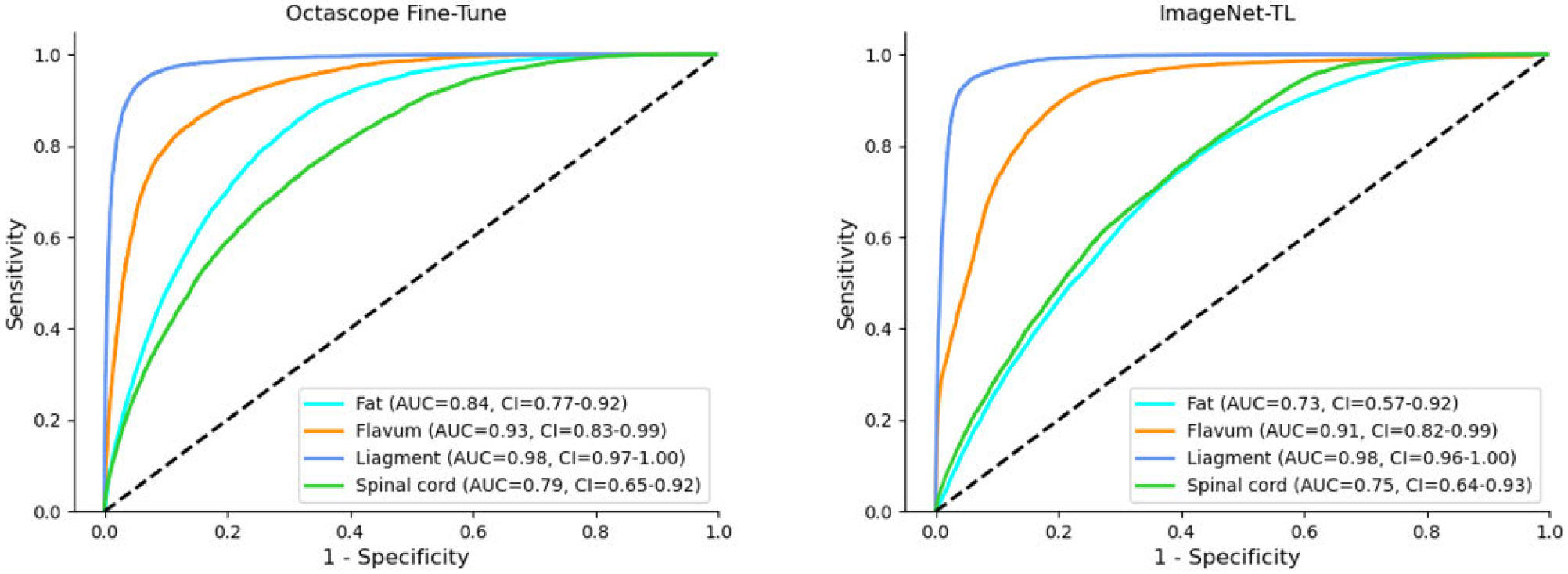
Average micro-ROC curves of five subjects’ cross-testing for fine-tuning with Octascope (left) and fine-tuning with ImageNet (right) with InceptionV3. The 95 Confidence interval (CI) was calculated by bootstrapping. The superior performance of Octascope shows that combining both OCT and natural image yields better results than relying solely on natural image features from ImageNet.

The ROC curves in Figure 4 demonstrated that the fine-tuned Octascope achieved better performance compared to the ImageNet-TL fine-tuned model across multiple tissue classes. Most notably, Octascope exhibited significantly better predictive capability for fat tissue, achieving an AUC of 0.84 compared to ImageNet-TL’s 0.73. Both models performed equally well in identifying ligament (AUC=0.98) and flavum (AUC=0.93) tissues, suggesting that these structures had distinctive features that could be reliably detected, regardless of the pre-training approach. Octascope maintained a slight advantage with an AUC of 0.79 versus ImageNet-TL’s 0.75 in detecting the spinal cord. These results indicated that Octascope’s domain-specific pre-training leads to more robust feature representation, particularly for challenging tissue types such as fat, which might share similar visual characteristics with the surrounding structures.

The performance of the subject-wise test varied significantly. For example, in the best-performing Octascope fine-tuned model, the difference between the highest performing subject (S4 with 73.68%) and the lowest (S3 with 56.23%) was 17.45%, suggesting high variability in tissue characteristics across subjects. This pattern of subject variability was consistent across different models, with subjects S6 and S7 generally achieving higher test accuracy, whereas subjects S2 and S3 consistently showed lower test accuracies among all base models, which were below 52.56% and 53.70% on average.

Table 2 presented the average confusion matrix comparing Octascope and RETFound predictions across eight subjects in this task. This confusion matrix was obtained, where each value was calculated by taking the average of the corresponding entries from individual confusion matrices generated for each fold during the CT loop of NCV. Octascope demonstrated notable improvements in flavum and spinal cord classification, correctly identifying an additional 24 and 25 cases, respectively, compared to RETFound. The ligament classification remained consistently high between both models (924 and 932 correct cases). The only exception was fat tissue classification, where RETFound showed marginally better performance than Otoscope, with 547 correct identifications under Octascope. These results suggested that Octascope’s pre-training strategy particularly enhanced the model’s ability to differentiate between similar tissue types, especially for the flavum and the spinal cord.

Octascope achieved remarkably faster training times, averaging 752.3 seconds compared to RETFound’s 4,265.2 seconds – with a speedup of approximately 5.7 folds as shown in Table 3. The training times remained consistent across all subjects, with Octascope ranging from 741 to 805 seconds, while RETFound required between 4,218.1 to 4,309.4 seconds. Even more significantly for practical clinical applications, Octascope’s inference consumed only 2.1 seconds per subject compared to RETFound’s 9.2 seconds - a 4.4 folds improvement in inference speed. This resulted in 0.75 milliseconds of latency per image on average with batch processing in predicting the tissue type within a subject. These results demonstrated that Octascope not only exceeded RETFound’s accuracy but also had significantly better computational efficiency, making it particularly attractive for real-world clinical applications where both accuracy and inference speed are crucial considerations.

The training process also showed significant efficiency gains with transfer learning enabled compared to STL: the total training time for eight subjects decreased from 7,017 seconds with random weight initialization in STL to 6,016 seconds with Octascope, saving 1,001 seconds while achieving a higher accuracy.

### B. COMPARISON OF MODELS FOR THE OCTDL RETINAL DIAGNOSIS TASK

Table 4 presented a comparison of the six different models for OCTDL retinal diagnosis. Given the consistent superiority of fine-tuning over feature extraction observed in previous experiments, we implemented only fine-tuning for Octascope and ImageNet-TL in this task. The baseline STL approach, training InceptionV3 from scratch with random initialization, achieved 79.18% accuracy, which demonstrated the challenge of learning robust features representation without any pre-training.

The OCTDL dataset exhibits notable class imbalance; for example, the RAO class contains only 22 samples. To mitigate the impact of this imbalance, we applied class reweighting based on the inverse frequency of training labels. This was implemented via the *class_weight* parameter in Keras during fine-tuning. As a result, Octascope achieved an improved accuracy of 92.02%, compared to 91.26% without class weights. This suggests that even simple imbalance-aware strategies can enhance performance, especially for rare disease categories.

The accuracy of Octascope with reweighting surpassed all baseline models in the retinal classification task. Specifically, it outperformed VGG16 and ResNet50 with TIMM pre-trained weights, which achieved accuracies of 85.9% and 84.6%, respectively. It also outperformed RETFound (90.79%) and ImageNet-TL (90.34%), showing the effectiveness of our curriculum-based pre-training strategy. The performance advantage of Octascope over Kulyabin et al.’s models [16]: improvements of 5.36% over VGG16 and 6.66% over ResNet50. It further validated the impact of using both natural and multi-tissue OCT data during pre-training. The consistent improvement of Octascope across both retinal disease diagnosis and epidural tissue detection demonstrates its potential as a domain-specific OCT foundation model, combining generalization and efficiency in a variety of clinical settings.

**FIGURE 5.**
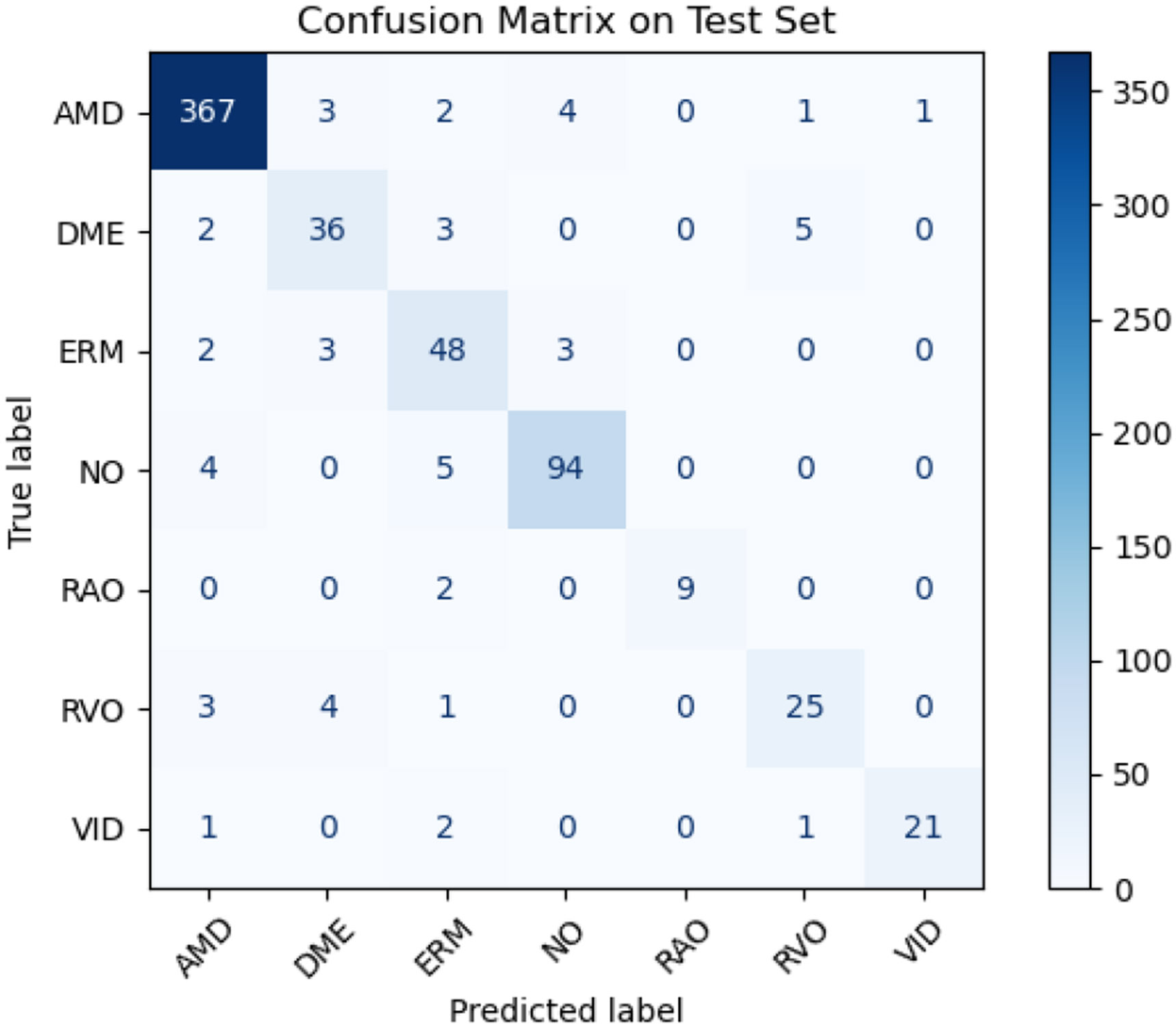
Confusion matrix of Octascope’s predictions on the test set of OCTDL. The matrix shows strong diagonal dominance with particularly high accuracy for AMD (367/378), Normal retina (94/103), and VID (21/25), demonstrating robust performance across both common and rare retinal conditions.

As shown in Figure 5, the confusion matrix reveals strong overall classification consistency. Notably, Octascope correctly predicted 9 of 11 RAO and 21 of 25 VID cases, indicating strong performance even in less represented classes. These trends are further quantified in Table 5, which produces high per-class $F_{1}$ scores for RAO (90.00%) and VID (89.36%). The consistent improvement of Octascope across both retinal disease diagnosis and epidural tissue detection demonstrates its potential as a domain-specific OCT foundation model, combining generalization and efficiency in a variety of clinical settings.

Octascope consumed only 180.3 seconds for training compared to RETFound’s 641.4 seconds - a 3.56 folds reduction in training time. It used just 1.1 seconds for inferencing on 652 images from the test set of the OCTDL dataset compared to RETFound’s 2.3 seconds. This demonstrated the superior computational efficiency of Octascope aligned with the results presented in the previous downstream task.

### C. ABLATION STUDY: IMPACT OF PRE-TRAINING AND CURRICULUM LEARNING

We conducted an ablation study to explicitly illustrate the incremental contributions of each design choice in Octascope’s pre-training approach. Specifically, we compared three pre-training strategies: (1) ImageNet-only pre-training (ImageNet-TL), (2) OCT-only pre-training (OCT-TL), and (3) curriculum learning (sequential pre-training on ImageNet followed by OCT datasets), represented by Octascope. Table 6 summarizes the comparative performance on two downstream tasks, epidural tissue detection and OCTDL retinal diagnosis, to clearly demonstrate the incremental gains from each training choice.

ImageNet-only pre-training establishes foundational visual features derived from natural images, which improves model performance compared to training from scratch. However, this approach lacks the domain-specific specialization required for precise OCT image classification tasks. OCT-only pre-training slightly outperforms ImageNet-only pre-training by 0.42%, as it directly captures relevant features from OCT data. Nonetheless, it does not benefit from the broader general visual knowledge that can aid representation learning. Curriculum learning, which involves sequential pre-training on ImageNet followed by OCT datasets, clearly yields superior accuracy. It outperforms ImageNet-only pre-training by 6.37% in the epidural tissue detection task and by 0.92% in the OCTDL retinal diagnosis task, while also surpassing OCT-only pre-training by 5.95%. These results confirm the effectiveness of curriculum learning in building both general and domain-specific representations for OCT analysis.

Curriculum learning explicitly enhances OCT diagnostic performance by addressing fundamental challenges in model optimization and data distribution [45], particularly when labeled data is limited. From an optimization perspective, it functions as a continuation method by simplifying the training objective in the early stages through exposure to general visual features derived from natural images. This approach helps the model converge toward better performing global minima rather than becoming cornered in suboptimal local minima. In terms of data distribution, curriculum learning simulates an incremental training strategy that begins with simpler, high-confidence subsets of the data. This structured progression supports more stable feature learning, and as more complex OCT-specific characteristics are introduced, the model becomes more robust to the noise and ambiguity often present in clinical datasets.

In addition to curriculum learning, we also considered architectural choices and training settings of CNN. We selected InceptionV3 as the backbone for Octascope based on its superior performance and efficiency in our fine-tuning experiments. For context, Table 4 includes performance results reported by the OCTDL study using ResNet50 under similar experimental settings. InceptionV3 achieved higher accuracy and significantly faster inference which supported its suitability for real-time clinical use. Furthermore, we found that freezing the early convolutional layers during the construction of Octascope led to slightly reduced performance. This observation suggests that full-model fine-tuning offers greater benefit for multi-domain pre-training.

## V. INTERPRETABILITY ANALYSIS

**FIGURE 6.**
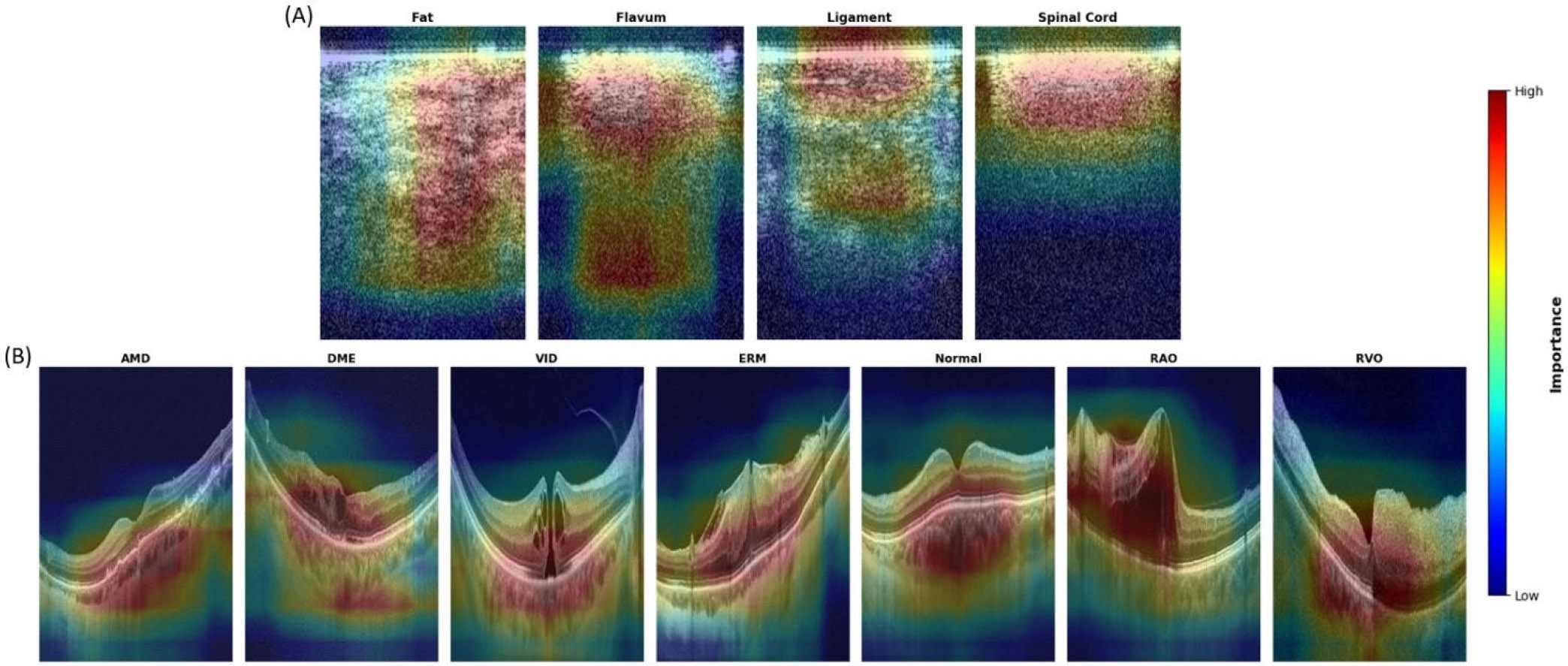
Heatmaps overlays generated with Grad-CAM of visualizations highlighting the class discriminative regions of two downstream tasks. (A) Epidural tissue detection across four tissue types. (B) OCTDL retinal diagnosis across seven conditions. Red regions indicate areas of highest model attention during the prediction. These visualizations validate Octascope’s learned feature representations and demonstrate its interpretability across different OCT imaging applications.

To further analyze and interpret the behavior of Octascope, we visualized heatmaps (Figure 6) highlighting the regions of interest during prediction. Specifically, we employed Grad-CAM [36] to generate class activation maps for the same sample images. These visualizations provide insights into the model’s attention patterns and help assess the alignment between its focus and relevant anatomical structures. In the epidural tissue detection task, we observed that the heatmaps for fat and flavum share relatively similar attention distributions, primarily covering broad and diffuse regions. This aligns with the lower predictive accuracy observed for these two classes, suggesting that their OCT image characteristics are less distinctive and more difficult for the model to differentiate. In contrast, the ligament and spinal cord displayed more localized and separable attention patterns, corresponding to clearer structural boundaries in the images. For the OCTDL retinal diagnosis task, the heatmaps highlight well-defined disease-associated regions across all disease and normal conditions, with distinct patterns observed for retinal abnormalities such as fluid accumulation, layer disruption, or tissue deformation. These observations are consistent with the predictive accuracy of over 90% achieved in this task, as the morphological features of each retinal disease are visually more distinguishable in OCT images.

## VI. DISCUSSION

Transfer learning from foundation models has emerged as a powerful approach in medical imaging, enabling improved model generalization across diverse clinical tasks with limited labeled data. Foundation models, pre-trained on large-scale datasets, capture fundamental visual patterns and domain-specific features that can be effectively transferred to downstream tasks through fine-tuning. Such models have demonstrated success across various medical imaging modalities, including MRI, X-ray, ultrasound, and OCT, achieving superior performance in classification, segmentation, and disease detection tasks. Unlike STL models trained from scratch, foundation models leverage prior knowledge to enhance predictive accuracy, accelerate convergence, and improve learning efficiency. In our study, Octascope, outperformed STL by 9.13% in the epidural tissue detection task and by 12.08% in the OCTDL retinal diagnosis task. Other base models, including ImageNet-TL, OCT-TL, VGG16 TIMM, and RETFound, also exhibited performance gains over STL, thereby reinforcing the effectiveness of transfer learning across different architectures and pre-training strategies. We systematically evaluated three key components for developing domain-specific foundation models in OCT image analysis: architecture selection, pre-training strategy, and downstream adaptation method.

First, we adopted a CNN-based architecture for Octascope. Compared to RETFound’s vision transformer (ViT) architecture, Octascope demonstrated significantly faster training and inference speeds while maintaining comparable accuracy. Specifically, the model achieved a 5.67-fold and 3.56-fold reduction in training time, and a 4.4-fold and 2.1-fold reduction in inference time for epidural tissue detection and OCTDL retinal diagnosis, respectively. This efficiency stems from architectural differences: Octascope, based on InceptionV3, contains 23.9 million parameters, occupies only 83.65 MB on disk, and requires approximately 8.15 GFLOPs per forward pass. In contrast, RETFound’s ViT-Large architecture comprises 307 million parameters and occupies 3.68 GB. This corresponds to a 92.21% and 97.70% reduction in parameter count and model size, respectively. Transformers such as RETFound incur additional computations owing to patch embedding, self-attention mechanisms, and masked autoencoder reconstruction [21], [22]. By contrast, CNNs exploit hierarchical local operations with shared weights, enabling efficient feature extraction and parameter reuse. Moreover, the architectural inductive bias of CNNs aligns well with the local structural patterns prevalent in medical images [23], making them particularly suitable for medical image analysis. While transformers capture long-range dependencies, this advantage may be less critical for OCT tasks, where local features are often sufficient for accurate predictions. ViTs may offer additional advantages in scenarios involving out-of-distribution (OOD) inputs, as their ability to aggregate global context can be valuable when dealing with novel disease manifestations or imaging artifacts. To further enhance Octascope’s adaptability in such settings, future versions could explore hybrid architectures that combine the computational efficiency of CNNs with the contextual modeling capacity of self-attention mechanisms during pre-training.

Second, we compared different pre-training strategies for model development. Octascope, pre-trained sequentially on natural images (ImageNet) followed by OCT images, demonstrated the strongest performance across downstream tasks. This supports the idea that curriculum learning, transferring knowledge from general to domain-specific data, can benefit medical imaging models. By acquiring general visual representations first, Octascope established a strong and transferable feature foundation that could be effectively adapted and refined for the unique characteristics of OCT imaging. This knowledge progression from broad to specific aligns with the principles of curriculum learning. A similar observation was made in RETFound, where pre-training on diverse ocular disease datasets led to better performance than using retinal images alone. RETFound outperformed SSL-Retinal in diabetic retinopathy classification across multiple datasets. Compared to RETFound, by incorporating OCT images from retinal, renal, and abdominal tissues during the pre-training stage, Octascope captured diverse anatomical structures and imaging characteristics from a variety of OCT images. This multi-domain exposure enhanced the performance of Octascope for both epidural tissue detection and OCTDL retinal diagnosis.

Third, we evaluated two downstream adaptation approaches. We observed that fine-tuning consistently outperformed feature extraction across all base models in the epidural tissue detection task. When employing feature extraction, in which only the classifier layers were trainable, the model was constrained to use fixed feature representations learned from the pre-training domain. In contrast, fine-tuning allowed the entire network to adapt its feature characteristics to the target domain through end-to-end training. The superiority of fine-tuning could be explained through the lens of CNN’s hierarchical feature learning. In CNNs, early layers typically learn basic visual patterns such as edges and textures, middle layers capture more complex patterns and shapes, while deeper layers learn domain-specific high-level features [26]. When all stages of layers were trainable, even these fundamental feature detectors in the early layers could be refined to better match the characteristics of medical images, which differed significantly from the natural images used in pre-training. Our findings on these two methods are aligned with observations of CADe [20]. In the study of Thoraco-abdominal lymph node (LN) detection, Shin. et al compared different transfer learning strategies using AlexNet. Their “off-the-shelf” method, which was the equivalent to our feature extraction, showed the poorest performance compared to fine-tuning and even models trained from scratch. The FROC curves for LN detection clearly proved that restricting adaptation to only the classifier layer significantly limited the model’s ability to effectively leverage the pre-trained ImageNet weights. These findings indicate that fine-tuning is more effective than feature extraction for downstream adaptation of pre-trained models by leveraging domain-specific refinements across all network layers.

Finally, we also explored progressive freezing as a transfer learning strategy for both downstream tasks. Specifically, we adopted a stage-wise unfreezing approach for the InceptionV3 backbone. Initially, we froze all convolutional layers and trained only the classification head. Subsequently, we incrementally unfroze the top layers, starting from the ‘mixed7’ and ‘mixed8’ blocks, which correspond to higher-level semantic features, followed by the unfreezing of intermediate layers such as ‘mixed5’ and ‘mixed6’. The lower layers remained frozen throughout, as these layers primarily captured the low-level visual patterns. However, the resulting performance was comparable to that of the standard feature extraction and full fine-tuning approaches, with no significant improvement observed. These findings suggest that, for our dataset and task complexity, progressive freezing provided limited additional benefit over conventional transfer learning methods.

As a 2D CNN-based model, Octascope was designed with computational efficiency and real-time clinical deployment in mind. This architectural choice allows for rapid inference and low memory usage, which are essential for time-sensitive clinical workflows. Octascope’s significant reduction in inference latency makes it particularly suitable for real-time clinical OCT applications. For example, during surgical guidance procedures such as epidural anesthesia needle placement [9] or intra-operative renal biopsies [11], the ability to rapidly classify tissues directly impacts patient safety and procedure accuracy. Faster inference speeds not only streamline the clinical workflow but also reduce the risk associated with delayed decision-making. Additionally, the lightweight architecture ensures compatibility with portable imaging systems and resource-constrained clinical environments. While Octascope operates on 2D slices, much of our pre-training data consists of slices extracted from volumetric data, enabling the model to learn a degree of structural variation across tissues. Future extensions of Octascope could explore 3D CNNs or hybrid models that integrate inter-slice information to capture volumetric context more directly, as demonstrated in recent 3D approaches like OCTCube and 3D-ViT [44].

From a clinical deployment perspective, predictive uncertainty estimation is a valuable capability. However, in this version of Octascope, we intentionally focused on maximizing inference efficiency and accuracy. Techniques such as Monte Carlo dropout [42] require multiple forward passes during inference, substantially reducing prediction speed, which is critical in time-sensitive clinical workflows. Furthermore, incorporating dropout layers may impair predictive accuracy. Similarly, deep ensembles [43] require multiple full training runs, which become computationally expensive, particularly for large-scale applications in medical imaging context. Given these trade-offs, we opted not to include uncertainty estimation in this version. Nonetheless, we recognize its potential benefits and consider it a promising direction for future iterations of Octascope that balance efficiency with model trustworthiness.

In terms of generalizability, Octascope has been pre-trained on a diverse multi-organ OCT dataset, which enhances its cross-tissue applicability. However, ongoing research efforts could further improve robustness under variations in OCT scanners, imaging protocols, or demographic groups. Employing domain generalization strategies or cross-site data harmonization techniques could be beneficial in this regard. Additionally, incorporating auxiliary imaging modalities, such as infrared reflectance (IR) or fundus photography [39], may provide complementary anatomical cues that strengthen the model’s diagnostic performance in ambiguous or subtle cases. For instance, recent work like OCTCube’s COIP framework illustrates how contrastive multi-modal pre-training can enhance cross-modality feature alignment and representation quality. Future versions of Octascope may similarly benefit from multi-modal fusion strategies, particularly for complex pathologies with systemic manifestations.

Our multi-domain pre-training strategy enhances generalization by exposing the model to a wide range of OCT image appearances and anatomical variability across different organ systems. This promotes domain-agnostic feature representations that allow Octascope to perform robustly even when deployed on previously unseen tissue types, as evidenced by its performance in the downstream tasks. Although the performance gains with Octascope may appear modest in absolute terms, they are clinically meaningful. For instance, an improvement of even 2-3% in tissue classification accuracy during image-guided procedures such as epidural anesthesia can reduce the risk of misidentification and improve the procedural outcomes. Combined with Octascope’s significantly lower inference latency and lightweight architecture, these accuracy gains translate into safer, faster, and more scalable deployment in real-word environments.

## VII. CONCLUSION

In this study, we introduced Octascope, a lightweight and performant domain-specific foundation model pre-trained on both ImageNet and diverse OCT datasets encompassing retinal, abdominal, and renal tissues. We evaluated Octascope on two representative OCT classification tasks: epidural tissue detection and retinal disease diagnosis using OCTDL. The results demonstrated that combining general visual knowledge from natural images with OCT imaging led to consistent performance improvements over traditional single-task learning and standard transfer learning strategies. Fine-tuning Octascope yielded the highest accuracy across both tasks, modestly outperforming both ImageNet-TL and OCT-TL baselines. Additionally, Octascope achieved slightly better predictive accuracy than RETFound while offering a significantly faster inference speed, making it a more efficient alternative for resource-constrained applications. These findings suggest that curriculum style pre-training across diverse data domains can enhance the adaptability of pre-trained models for OCT image analysis. Although the observed gains were incremental, they indicate a valuable direction for developing lightweight and generalizable foundation models in medical imaging.

## Figures and Tables

**FIGURE 1.**
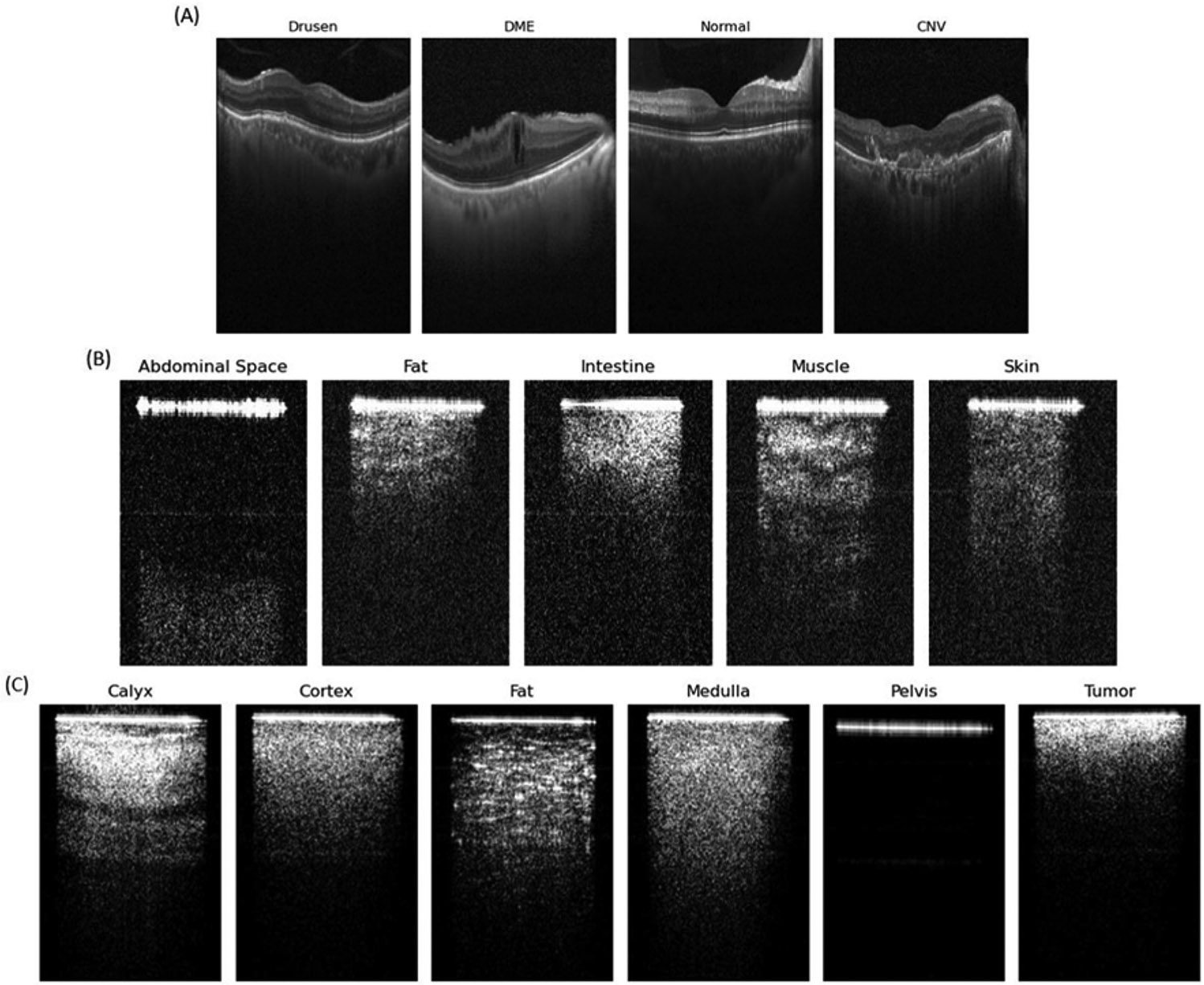
Example OCT images of a variety of tissues from the three pre-training datasets. (A) Retinal OCT images showing various conditions: Drusen, DME (Diabetic Macular Edema), Normal retina, and CNV (Choroidal Neovascularization). (B) Veress needle OCT images of five different abdominal tissues: Abdominal Space, Fat, Intestine, Muscle, and Skin. (C) Human kidney images from various renal tissues: Calyx, Cortex, Fat, Medulla, Pelvis, and Renal tumor. The distinct visual characteristics make it easy to differentiate between the two types of fat tissue: human kidney fat appears as bright dots typical of adipocytes, while fat in the Veress Needle dataset displays granular structures.

**FIGURE 2.**
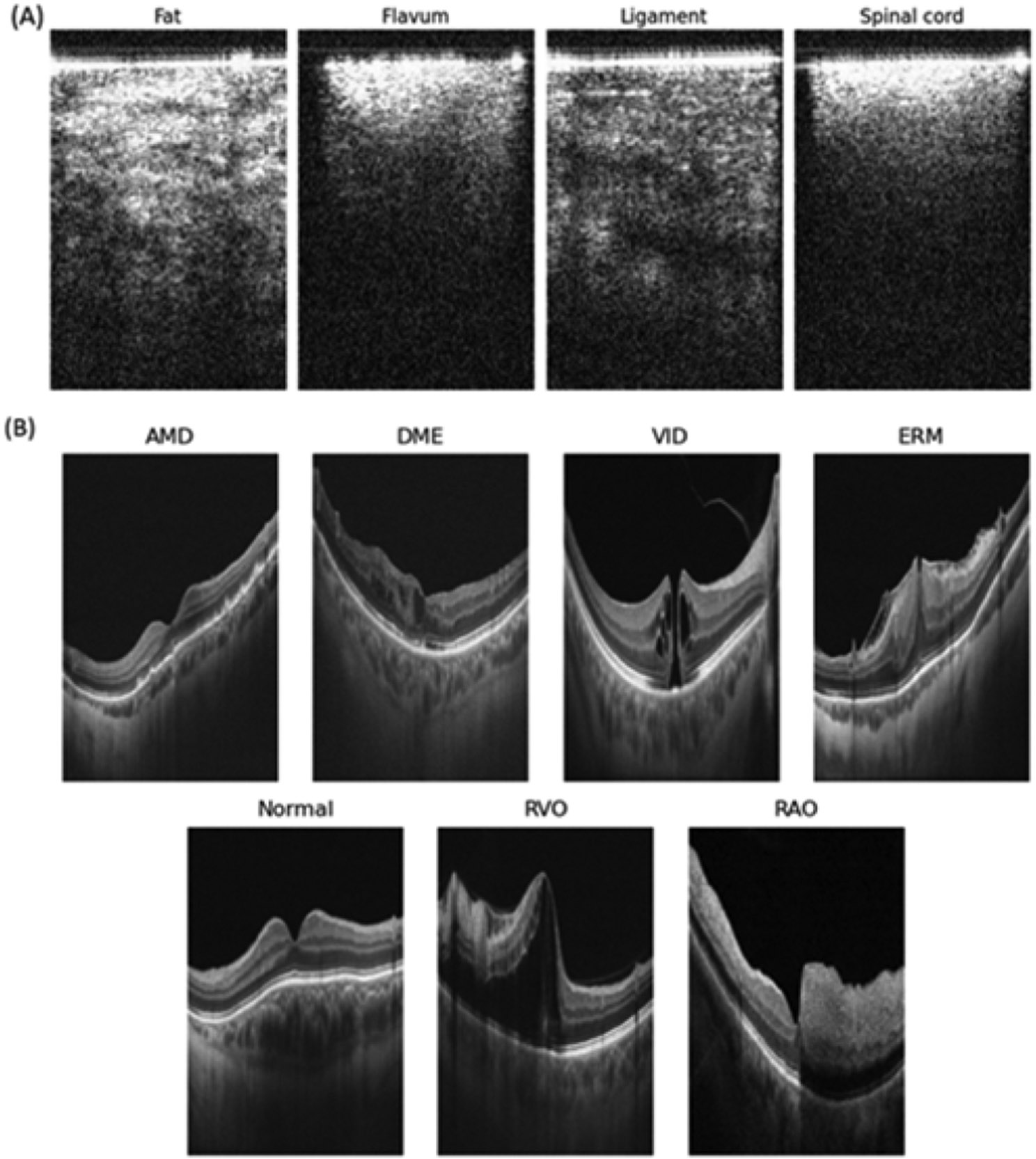
Spinal tissues and retina of various conditions from the two downstream tasks. (A) The epidural tissue detection task. Example OCT images were shown for Fat, Flavum (ligamentum flavum), Ligament, and Spinal cord from porcine spines. The different OCT features of spinal tissues can be used to localize a needle in real time during needle placement for epidural anesthesia. (B) The OCTDL retinal diagnosis task. Example OCT images were shown for human retina of different conditions, including AMD, DME, VID, ERM, Normal retina, RVO, and RAO. The OCT images captured varied presentations of retinal pathologies and normal anatomy. These examples demonstrate the diverse visual presentations that OCT captures across different clinical contexts.

**TABLE 1.** Performance benchmarking results for the epidural tissue detection task.

Base Model	Adaptation	Test Accuracy by Subjects (percentage)								
		S1	S2	S3	S4	S5	S6	S7	S8	Avg±SE
STL	TN	56.55	54.37	53.00	66.75	58.40	63.37	62.08	61.18	59.50±1.67
ImageNet-TL	FE	63.80	47.53	52.53	67.12	63.52	60.05	69.92	62.52	60.87±2.63
	FT	53.10	54.65	50.08	69.55	66.75	79.87	60.15	63.95	62.26±3.49
OCT-TL	FE	61.83	44.03	48.45	54.83	58.15	64.23	67.22	63.08	57.72±2.87
	FT	66.28	57.13	50.05	60.30	59.25	69.73	70.76	67.84	62.68±2.54
**RETFound**	FT	71.25	55.53	58.67	72.15	63.90	75.58	72.47	70.83	**67.55±2.57**
**Octascope**	FE	66.83	47.35	60.58	64.35	64.40	66.10	69.62	66.80	63.25±2.45
	FT	71.22	59.85	56.23	73.68	62.25	79.28	76.59	69.92	**68.63±2.93**

**TABLE 2.** Average confusion matrix for octascope and retfound on the epidural tissue detection task.

Ground Truth	Prediction (Octascope)				Prediction (RETFound)			
	Fat	Flavum	Ligament	Spinal cord	Fat	Flavum	Ligament	Spinal cord
Fat	**547**	54	86	314	**563**	20	75	342
Flavum	46	**750**	29	174	64	**726**	13	198
Ligament	65	8	**924**	4	51	5	**932**	13
Spinal cord	262	170	19	**512**	367	130	16	**487**

**TABLE 3.** Average Training and Inference Time in CT Loop of NCV with Octascope and RETFound.

Base Model	Stage	Time (seconds)
Octascope	Training	752.3
	Inference	2.1
RETFound	Training	4265.2
	Inference	9.2

**TABLE 4.** Prediction accuracies, training, and inference speed of OCTDL retinal diagnosis.

Base Model	Accuracy	Training time (seconds)	Inference time (seconds)
VGG16	85.9%	N/A	N/A
ResNet50	84.6%		
STL	79.18%	211.1	1.4
ImageNet-TL	90.34%	179.1	1.2
RETFound	90.79%	641.4	2.3
Octascope	91.26%	180.3	1.1
**Octascope-reweighting**	**92.02%**	**181.5**	**1.1**

**TABLE 5.** OCTDL’s Per-class precision, recall, and $F_{1}$ score for Octascope-reweighting on test set.

Class	Precision	Recall	$F_{1}$ score
**AMD**	96.83%	97.09%	**96.96%**
**DME**	78.26%	78.26%	**78.26%**
**ERM**	76.19%	85.71%	**80.67%**
**NO**	93.07%	91.26%	**92.16%**
**RAO**	100%	81.82%	**90.00%**
**RVO**	78.13%	75.76%	**76.93%**
**VID**	95.46%	84.00%	**89.36%**
**Macro-Average**	N/A	N/A	**86.33%**

**TABLE 6.** Performance comparison of pre-training strategies in two downstream tasks.

Pre-training strategy	Epidural tissue detection	OCTDL retinal diagnosis
**ImageNet-TL (ImageNet)**	62.26%	90.34%
**OCT-TL (OCT)**	62.68%	N/A
**Octascope (ImageNet + OCT)**	68.63%	91.26%
